# Knowledge, skills, and barriers to management of faecal incontinence in Australian primary care: a cross-sectional study

**DOI:** 10.3399/BJGPO.2020.0182

**Published:** 2021-04-21

**Authors:** Kheng-Seong Ng, Deanne S Soares, Sireesha Koneru, Ramesh Manocha, Marc Antony Gladman

**Affiliations:** 1 The Bowel Clinic, Adelaide, Australia; 2 Sydney Medical School, University of Sydney, Sydney, Australia; 3 University Hospital, Geelong, Australia; 4 HealthEd / Generation Next, Sydney, Australia; 5 Adelaide Medical School, Faculty of Health & Medical Sciences, The University of Adelaide, Adelaide, Australia

**Keywords:** faecal incontinence, primary health care, general practice, referral pathways

## Abstract

**Background:**

GPs play an important role in the diagnosis and management of patients with faecal incontinence (FI). However, their confidence and ability in this role are unknown.

**Aim:**

This study aimed to investigate the knowledge, skills, and confidence of GPs to manage FI in primary care, and identify barriers to optimal management.

**Design & setting:**

A cross-sectional study using self-administered questionnaires of GPs attending health education seminars, which took place across Australian capital cities.

**Method:**

Main outcome measures included: (i) clinical exposure to and previous training in FI; (ii) knowledge and skills in screening, diagnosing, and managing FI; and (iii) barriers and facilitators to optimising care. Associations between demographics, training and knowledge and skills were assessed.

**Results:**

Some 1285 of 1469 GPs (87.5%) participated (mean 47.7 years [standard deviation {SD} 11.3]). The vast majority reported poor clinical exposure to (88.5%) and training in FI management (91.3%). Subjectively, 69.7% rated their knowledge and skills in screening, assessing, and treating FI as suboptimal. The most commonly reported barrier to FI care was ‘insufficient skills’ (56.1%); facilitators were improved referral pathways (84.6%) and increased training (67.9%). GPs with more training had better knowledge (odds ratio [OR] = 24.62, 95% confidence interval [CI] = 13.32 to 45.51) and skills (OR = 13.87, 95% CI = 7.94 to 24.24) in managing FI.

**Conclusion:**

Clinical exposure to and training in FI among GPs was poor. Accordingly, knowledge, skills, and confidence to manage FI was suboptimal. GPs recognise the importance of FI and that increased training and/or education and formalisation of referral pathways may improve the care of patients with FI in primary care.

## How this fits in

The role of primary health care in the management of FI is increasingly recognised, but the clinical exposure and training and/or education that primary healthcare professionals (GPs) receive for this condition is unknown. This study identified that: (i) the majority of GPs report poor clinical exposure to and training in FI management; (ii) the most commonly reported facilitators to FI care include improved referral pathways and increased training; (iii) GPs with more previous training have better skills in managing FI; and (iv) increased training and/or education and formalisation of referral pathways may facilitate primary health care of FI, and and optimise interaction with secondary care providers.

## Introduction

FI affects approximately 12% of adults in the community,^1^ making it more prevalent than diabetes mellitus (4.9%), osteoporosis (3.8%), and cancer (1.8%) combined.^2^ It impacts on general health, as well as emotional and mental wellbeing. It also socially isolates sufferers, increases healthcare costs,^3^ and is the second leading cause for nursing home placement.^4^ Effective management thus provides an important public health opportunity to keep patients in the community, improve health, and reduce the burden on healthcare resources.

Traditionally, the management of FI has been considered the work of the specialist. While this is appropriate for patients who fail to respond to conservative measures, the importance of detection and initiating appropriate management in primary care has increasingly been recognised.^5–7^ Over 12% of patients availing themselves to primary care have FI,^8^ and GPs occupy a critical position in the patient care pathway with respect to detection, initial management, and referral when simple interventions fail to alleviate symptoms. To be effective in this role, GPs need to be equipped with the necessary knowledge and skills to manage FI, which is challenging as FI has been termed *'* *a silent affliction* *'*, as it is frequently not volunteered by patients owing to embarrassment.^9^ However, the clinical exposure and amount of training GPs receive on this important topic has not been previously investigated. Therefore, this study aimed to: (i) investigate the attitudes and beliefs of Australian GPs regarding FI; (ii) to assess the clinical exposure, knowledge, and skills of GPs to diagnose and manage FI at a primary care level; and (iii) explore what GPs perceive to be challenges and barriers to the optimal management of FI in primary care.

## Method

### Study design, setting, and population

A cross-sectional study was performed of GPs attending health education seminars focused on women’s health during 2016–2017 across major Australian cities (Sydney, Melbourne, Brisbane, Adelaide, and Perth). All GPs who attended were invited to complete a self-administered written questionnaire prior to education being delivered. As the study was not a comparative interventional study, a formal power calculation was not performed. However, a minimum sample size of 1000 participants was based on a previous study.^6^

### Questionnaire

The questionnaire comprised 22 items (see Supplementary Box 1), including demographic information relating to age, sex, practice location, number of years’ clinical experience in primary health care, and area of clinical interest. Subsequent questions relating to FI focused on four themes:

- Practical and theoretical training
  - Clinical exposure and previous education in FI was ascertained via questions including: 'What proportion of your patients present with bowel leakage?'; and 'How much training have you received for managing FI?'
- Theoretical knowledge
  - Self-rated knowledge was assessed using questions such as: 'How would you rate your knowledge (overall and of available surgical procedures) of FI?'; and
  - the accuracy of responses to questions such as: 'What is the prevalence of FI among primary healthcare seekers?'; and 'What are the risk factors or appropriate investigations in patients with FI?'
- Clinical skills in the screening, assessment, and first-line treatment of FI
  - Information was obtained from the responses to questions including: 'Which tests would you organise for patients with FI?'; and 'What degree of confidence do you have initiating first-line treatments?'
- Barriers to and facilitators of optimal FI management in primary care
  - Sought by asking GPs to identify pertinent factors from a list of options.

Most questions employed a 5-point scale (very poor, poor, reasonable, good, very good) for response options. The questionnaire was developed and revised by senior clinicians in primary and secondary care, and pilot tested on 'lay’ individuals. The pilot-testing process obtained feedback regarding question clarity and validity and were revised (or excluded), as necessary.

### Statistical analyses

Data were analysed using frequency tabulations and contingency tables. Where appropriate, participant characteristics and responses were dichotomised by collapsing responses (that is, ‘very poor’ or ‘poor’, and ‘reasonable’ to ‘very good’) for the analyses or by selecting suitable thresholds a posteriori. Associations between various demographic characteristics, level of previous training or education, and knowledge and skills in FI management were assessed by χ^2^ analysis, and presented as crude OR with 95% CIs. These were of particular interest to establish whether training resulted in better skills and/or knowledge, with a view to identifying interventions for future studies. Missing data were treated by complete case analysis; no imputation methods were used as doing so could have introduced undue bias. All analyses were conducted using Stata (version 15). *P*<0.05 was considered statistically significant.

## Results

Of 1469 GPs who attended the seminars, 1285 (87.5%) participated in the study (mean age 47.7 years, SD 11.3). Demographic details are presented in Table 1 and supplementary Table S1. Notably, 80.2% (*n* = 894) of GPs were female, the majority (61%, *n* = 650) practised in a metropolitan (urban) location, and 27.5% (*n* = 218) had more than 20 years’ experience. The main areas of clinical interest included women’s health (*n* = 716, 64.0%) and paediatrics (*n* = 397, 35.5%).

**Table 1.** Demographic details and clinical exposure and previous training or education in managing FI

Demographics	*n* (%)
Sex	
Male	221 (19.8)
Female	894 (80.2)
Practice location	
Metropolitan	650 (61.0)
Regional	372 (34.9)
Remote	44 (4.1)
Specific area(s) of clinical interest	
Women’s Health	716 (64.0)
Antenatal care	361 (32.3)
Dermatology	208 (18.6)
Aged care	178 (15.9)
Paediatrics	397 (35.5)
Other	201 (18.0)
No specific area of clinical interest	232 (20.7)
**Clinical exposure, previous training** **,** **or education in managing FI**	
Training or education received for FI management	
None	406 (32.0)
Small amount	752 (59.3)
Moderate amount	97 (7.6)
Substantial amount	10 (0.8)
Large amount	4 (0.3)
Training or education received for UI management	
None	53 (4.2)
Small amount	670 (52.5)
Moderate amount	471 (36.9)
Substantial amount	74 (5.8)
Large amount	8 (0.6)
Training or education received for managing ‘bowel problems’	
None	129 (10.2)
Small amount	609 (48.0)
Moderate amount	442 (34.8)
Substantial amount	81 (6.4)
Large amount	9 (0.7)
GP wishing to receive more training and/or education	
No	65 (5.1)
Yes	1206 (94.9)
By face-to-face lectures	606 (50.2)
By online courses	502 (41.6)
By reading material	325 (26.9)
By DVD	175 (14.5)

Totals for individual items may not equal 1285 owing to missing data. FI = faecal incontinence. UI = urinary incontinence.

### Practical and theoretical training

As presented in Table 1, managing patients with FI made up less than 5% of the GP’s workload for the vast majority (*n* = 1127, 88.5%). Only 8.6% (*n* = 111) had received a moderate, substantial, or large amount of training and/or education specific to the management of FI, compared with 43.3% (*n* = 553) for management of urinary incontinence and 41.9% (*n* = 532) for bowel problems in general (Table 1). Almost all (*n* = 1206, 94.9%) wanted more training, with interactive face-to-face lectures being preferred (*n* = 606, 50.2%). Additional data are presented in Supplementary Table S1.

### Knowledge

Most (69.7%, *n* = 885) GPs self-rated their overall knowledge as ‘very poor’ or ‘poor’ (Table 2). Objectively, the prevalence of FI among primary healthcare seekers was correctly determined by only 17.2% (*n* = 217), although the majority correctly identified important risk factors for FI, including neurological or spinal conditions, a history of anal surgery and obstetric trauma (*n* = 983, 76.7%) (Table 2). With respect to investigations, the majority recognised the importance of digital rectal examination (*n* = 1003, 79.0%) and colonoscopy (*n* = 825, 65.0%), but less than one-half recognised the importance of anal manometry (*n* = 531, 41.8%) or endoanal ultrasound (*n* = 283, 22.3%) (Table 2). Additional data relating to knowledge are presented in Supplementary Table S2.

**Table 2.** Knowledge and skills of GPs regarding FI

Knowledge	*n* (%)
GP’s self-rated overall knowledge about FI	
Very poor	206 (16.2)
Poor	679 (53.5)
Reasonable (neither poor nor good)	365 (28.7)
Good	17 (1.3)
Very good	3 (0.2)
GP’s estimation of FI prevalence in primary care	
<1%	60 (4.8)
1%–4%	457 (36.2)
5%–9%	406 (32.2)
10%–14%	217 (17.2)
15%–24%	100 (7.9)
≥25%	21 (1.7)
Which investigation(s) GP would arrange to investigate FI	
Faecal occult blood testing	556 (43.8)
Colonoscopy	825 (65.0)
Abdominal X-ray	272 (21.4)
Abdominal ultrasound	175 (13.8)
Endoanal ultrasound	283 (22.3)
Computed Tomography (CT) scan abdomen or pelvis	216 (17.0)
Anal manometry	531 (41.8)
Digital rectal examination	1003 (79.0)
**Skills**	
GP’s self-rated overall skills in treating patients with FI	
Very poor	141 (11.1)
Poor	713 (56.1)
Reasonable (neither poor nor good)	396 (31.2)
Good	19 (1.5)
Very good	2 (0.2)
GP’s self-rated confidence in initiating lifestyle or conservative measures for patients with FI	
Very poor	152 (12.0)
Poor	288 (22.7)
Reasonable (neither poor nor good)	320 (25.2)
Good	408 (32.1)
Very good	102 (8.0)
GP’s self-rated confidence in prescribing medication(s) to treat FI	
Very poor	284 (22.3)
Poor	369 (29.0)
Reasonable (neither poor nor good)	324 (25.5)
Good	267 (21.0)
Very good	28 (2.2)

Totals for individual items may not equal 1285 owing to missing data. FI = faecal incontinence.

### Skills

The majority of GPs rated their skills in screening, assessing, and treating patients with FI to be ‘very poor’ or ‘poor’ (Table 2 and Supplementary Table S2). Although 40.1% (*n* = 510) rated their confidence to initiate lifestyle or conservative measures as ‘good or very good’, only 23.2% (*n* = 295) were confident prescribing medication ('good or very good') (Table 2). The majority (*n* = 910, 72.6%) would refer patients with FI to a colorectal surgeon for further specialist management (see Supplementary Table S2).

### Barriers and facilitators

Potential barriers and facilitators to screening and treating patients with FI are presented in Table 3. The most commonly reported barriers were ‘insufficient skills’ (*n* = 703, 56.1%) or concerns that patients may not be receptive (*n* = 378, 30.1%) (Table 3). The most commonly reported facilitators for improved management in primary care were knowing exactly where (*n* = 938, 74.1%) and to whom (*n* = 1071, 84.6%) to refer to, and training (*n* = 860, 67.9%).

**Table 3.** Barriers and facilitators in management of FI in primary care

Barriers to screening and treating patients with FI	***n* (%)**
Insufficient skills	703 (56.1)
FI not common or significant enough to justify enquiring with patient	95 (7.6)
Concerns that patient may not be receptive to screening or intervention	378 (30.1)
Insufficient time to screen or provide intervention	150 (12.0)
Insufficient support from specialists	178 (14.2)
Wish to avoid further referrals of patients with FI in the future	38 (3.0)
Perception that FI has no effective treatment, so screening is futile	83 (6.6)
Perception that FI is not the most important issue during the consultation	115 (9.2)
Lack of interest in screening or treating FI	70 (5.6)
GP’s embarrassment to ask patients about any bowel leakage	50 (4.0)
Avoidance of patient’s embarrassment if probed about bowel leakage problems	113 (9.0)
Perception that FI should only be treated by specialists in the field	76 (6.1)
**Facilitators to screening and treating patients with FI**	
Knowing exactly who to refer to	1071 (84.6)
Knowing exactly where to refer to	938 (74.1)
Easier referral pathway	583 (46.1)
More resources to assist	677 (53.5)
Having effective treatments available	567 (44.8)
Belief among GPs that screening and intervention are important	590 (46.6)
Having more detailed communication from specialists after referral	609 (48.1)
Having less detailed communication from specialists after referral	75 (5.9)
Access to up-to-date management guidelines and recommendations	898 (70.9)
Further training to allow GP to be more comfortable talking to patients about FI	570 (45.0)
Further training to allow GP to be more knowledgeable treating FI	860 (67.9)
No facilitators identified	42 (3.3)

Totals for individual items may not equal 1285 owing to missing data. FI = faecal incontinence.

### 

### Factors associated with practical and theoretical training in managing FI

GPs aged >50 years, those who practised in regional or remote locations, those in clinical practice for >15 years, and those with an interest in aged care were more likely to have greater clinical exposure to patients with FI (comprising >5% of their workload). GPs who had a ‘moderate’ to ‘large’ amount of previous training and education in the management of FI were more likely to see a greater proportion of patients with FI, and more likely to have an interest in aged care (see supplementary Table S3).

### Factors associated with knowledge and skills in managing FI

GPs who received substantial training in FI were almost 25 times more likely to self-rate their overall knowledge of FI higher (OR = 24.62, 95% CI = 13.32 to 45.51, *P*<0.001) and 14 times more likely to self-rate skills in screening, assessing, and treating FI higher (screening and assessing: OR = 13.87, 95% CI = 7.94 to 24.24; and treating FI: OR = 13.21, 95% CI = 7.85 to 22.26, *P*<0.001). GPs with an interest in aged care (OR = 1.73, 95% CI = 1.24 to 2.41) and those with greater clinical exposure (OR = 2.49, 95% CI = 1.75 to 3.53) (self-)rated their knowledge and skills higher (*P*<0.001).

Factors associated with greater confidence of skills treating FI in are shown in Table 4. Specifically, GPs working in regional or remote locations and those who had been in clinical practice for >15 years were significantly more likely to report greater confidence in initiating lifestyle measures and prescribing medications for FI, as well as better knowledge of surgical procedures for FI (see Table 4). Similarly, GPs with an interest in aged care, those with greater clinical exposure, and those having received a moderate to large amount of previous training or education in FI were more confident treating patients with FI.

**Table 4.** Factors associated with confidence in treating patients with FI

	Confidence to initiate lifestyle or conservative measures	Confidence to prescribe medications for FI	Self-rated knowledge of surgical procedures for FI
	Very poor or poor	Reasonable or very good	OR (95% CI)	Very poor or poor	Reasonable or very good	OR (95% CI)	Very poor or poor	Reasonable or very good	OR (95% CI)
**Practice location**									
Metropolitan	240 (65.6%)	402 (58.4%)		357 (64.3%)	286 (57.1%)		386 (63.7%)	252 (57.1%)	
Regional or remote	126 (34.4%)	287 (41.7%)	1.36 (1.04 to1.77)^a^	198 (35.7%)	215 (42.9%)	1.36 (1.06 to 1.74)^a^	220 (36.3%)	189 (42.9%)	1.32 (1.02 to 1.69)^a^
**Years in clinical practice**									
≤15 years	160 (58.8%)	258 (50.3%)		242 (57.9%)	176 (48.0%)		258 (57.1%)	157 (47.7%)	
>15 years	112 (41.2%)	255 (49.7%)	1.41 (1.05 to 1.90)^a^	176 (42.1%)	191 (52.0%)	1.49 (1.13 to 1.98)^b^	194 (42.9%)	172 (52.3%)	1.46 (1.10 to 1.94)^b^
**Special clinical interest**									
None	95 (25.1%)	135 (18.5%)	0.68 (0.50 to 0.91)^a^	123 (21.4%)	107 (20.0%)	0.91 (0.68 to 1.22)	140 (22.3%)	88 (18.8%)	0.81 (0.60 to1.09)
Women’s health	224 (59.1%)	484 (66.3%)	1.36 (1.05 to 1.76)^a^	370 (64.5%)	339 (63.3%)	0.95 (0.74 to 1.21)	391 (62.2%)	314 (67.0%)	1.23 (0.96 to 1.58)
Antenatal care	126 (33.3%)	233 (31.9%)	0.94 (0.72 to 1.23)	193 (33.6%)	166 (31.0%)	0.89 (0.69 to 1.14)	203 (32.3%)	150 (32.0%)	0.99 (0.76 to 1.27)
Dermatology	59 (15.6%)	148 (20.3%)	1.38 (0.99 to 1.92)	98 (17.1%)	109 (20.3%)	1.24 (0.92 to 1.68)	112 (17.8%)	93 (19.8%)	1.14 (0.84 to 1.55)
Aged care	40 (10.6%)	138 (18.9%)	1.98 (1.36 to 2.88)^c^	74 (12.9%)	104 (19.4%)	1.63 (1.18 to 2.25)^b^	91 (14.5%)	85 (18.1%)	1.31 (0.95 to 1.81)
Paediatrics	131 (34.6%)	262 (35.9%)	1.06 (0.82 to 1.37)	216 (37.6%)	178 (33.2%)	0.82 (0.64 to 1.05)	217 (34.5%)	175 (37.3%)	1.13 (0.88 to 1.45)
**Clinical exposure (percentage of workload comprising patients with FI)**									
<5%	403 (92.2%)	711 (86.3%)		597 (92.1%)	519 (84.4%)		657 (92.2%)	449 (83.8%)	
≥5%	34 (7.8%)	113 (13.7%)	1.88 (1.26 to 2.82)^b^	51 (7.9%)	96 (15.6%)	2.17 (1.51 to 3.10)^c^	56 (7.9%)	87 (16.2%)	2.27 (1.59 to 3.25)^c^
**Training or education in FI management**									
None or small amount	418 (96.5%)	728 (88.5%)		619 (96.4%)	529 (85.9%)		681 (96.1%)	454 (84.9%)	
Moderate or large amount	15 (3.5%)	95 (11.5%)	3.64 (2.08 to 6.35)^c^	23 (3.6%)	87 (14.1%)	4.43 (2.76 to 7.11)^c^	28 (4.0%)	81 (15.1%)	4.34 (2.78 to 6.78)^c^

^a^ *P*<0.05; ^b^ *P*<0.01; ^c^ *P*<0.001. FI = faecal incontinence.

## Discussion

### Summary

This large study of skills, knowledge, and barriers to the management of FI by GPs revealed that education, training, and clinical exposure to this condition was poor, and that most rated their knowledge and skills in screening, assessing, and treating FI as suboptimal. Nevertheless, the majority of GPs were keen to embrace their role in detecting and managing this condition; however, clear barriers were identified that need to be addressed to optimise management at a primary care level, especially the inadequacy of prior training or experience among GPs. This study also confirmed the positive impact that training and education has in improving the knowledge, skills, and confidence of GPs to detect and manage FI.

### Strengths and limitations

This study was strengthened by the large number of participants captured across a multi-centre, nationwide audience and a large response rate. However, it was limited by the convenience sampling as the study population included GPs attending educational activities and, thus, may be considered a highly motivated audience. Further, there was a sex bias, with over 80% being female. Finally, while there were obvious positive relationships between previous training and self-rated knowledge, skills, and confidence in FI management, this study was not able to investigate whether the same was true for objective evaluation of knowledge and skills.

### Comparison with existing literature

Approximately 90% of GPs reported that patients with FI constituted <5% of their workload, despite previous studies demonstrating that approximately 12% of primary healthcare seekers admitted to suffering with FI when directly asked.^8,10^ This discrepancy confirms the reluctance of sufferers to volunteer symptoms owing to stigma that surrounds the condition,^9^ leading to suboptimal detection of FI unless specifically sought during consultations.^11^ Indeed, cases of FI detected in the primary healthcare system in a study of over 65 000 participants suggested a prevalence of only 0.1% whereas the actual prevalence in the population is 13%.^12^ Further, >90% of patients with FI waited at least 1 year before reporting their symptoms and all patients reported that their healthcare provider had not specifically asked them about this problem.^12^

This study emphasised current inadequacy in training and education for FI management in primary care, as has previously been reported.^13^ The overwhelming majority of GPs reported either ‘no’ or ‘a small amount’ of prior training. Consequently, it is of no surprise that over two-thirds rated their overall knowledge of FI to be ‘very poor or poor’, corroborated by the fact that only one in six knew the correct prevalence of FI among primary healthcare seekers, and over half failed to recognise the importance of loose stool consistency as a risk factor for FI.^10,14^ However, the majority recognised other important risk factors for FI, including neurological or spinal conditions,^15^ and previous obstetric trauma.^16^ By comparison, less than one-half appreciated the importance of investigations such as anal manometry and endoanal ultrasound in the assessment of FI,^17^ a finding consistent with a previous study in which only 32% of GPs were aware of one or more investigations.^6^

These findings provide an important educational opportunity, particularly as over two-thirds of GPs felt that further training in treating FI would facilitate patient care, and 95% o indicated a desire to receive more training, citing a clear preference towards interactive face-to-face lectures rather than self-directed learning. The United Kingdom Continence Society has recommended explicit minimum standards for structured training of clinicians as part of minimum standards for continence care.^18^ Currently, curricula from the Royal Australian College of General Practitioners (RACGP) do not mention FI within their core skills units.^19,20^ This is in stark contrast to training in urinary incontinence, with over 40% of GPs reporting a ‘moderate’ to ‘large’ amount of training on this topic. This disparity probably explains why GPs demonstrate readiness to screen for urinary incontinence but not FI.^11^ Indeed, the current RACGP Guidelines for Activities in General Practice dedicates an entire chapter to the topic of urinary incontinence, but makes no reference to FI.^21^ Current FI management resources available from the RACGP are targeted at aged care patients in residential institutions.^22,23^ The presumed association between FI and aged care may explain why GPs with a specialty interest in aged care had better self-rated knowledge, skills, and confidence to manage FI in the study.

### Implications for research and practice

GPs recognised that FI is an important chronic condition with effective treatment options, but felt ill-equipped and under-trained to contribute effectively to patient management. Importantly, GPs who reported having received a ‘moderate’ to ‘large’ amount of previous training or education had substantially increased odds of better knowledge, skills, and confidence in managing FI, which was independent of clinical exposure to patients with FI and years working in primary care. Additionally, there was an association between previous training and ongoing clinical exposure to the condition. Specifically, GPs who reported that more than 5% of their workload was made up of patients with FI had five-fold increased odds of having had a ‘moderate’ to ‘large’ amount of previous training. While this could reflect the fact that these GPs pursued self-directed training to better equip themselves to deal with this condition, it may also reflect an increased awareness to actively screen for symptoms of FI. Such a proactive approach at a primary care level has been demonstrated to significantly increase rates of detection by over five-fold,^24^ and could be expected to result in earlier diagnoses and, thus, greater clinical exposure to the condition.

GPs identified important potential facilitators to optimise the management of FI, including improved referral pathways to secondary and tertiary care providers, and more detailed communication from specialists after referral. There was uncertainty about knowing exactly who and where to refer a patient with FI, consistent with previous studies.^6^ GPs also felt that accessibility to specialist care was limited.^25^

In conclusion, the vast majority of Australian GPs have received no specific training in the management of FI and thus, unsurprisingly, knowledge, skills, and confidence to manage patients with FI was suboptimal. There was recognition among GPs that management of FI in primary care is important, and increased training and education, and formalisation of referral pathways were considered the most important facilitators to improve care of patients with FI. Whether such measures can keep patients in the community, improve their health, and reduce the burden on community and government healthcare resources warrants further investigation.

## Trial registration number

N/A

## References

[1] Ng K-S, Sivakumaran Y, Nassar N, Gladman MA (2015). Fecal incontinence: community prevalence and associated factors—a systematic review. Dis Colon Rectum.

[2] Australian Bureau of Statistics (2018). National health survey: first results, 2017–18. https://www.abs.gov.au/ausstats/abs@.nsf/mf/4364.0.55.001.

[3] Dunivan GC, Heymen S, Palsson OS (2010). Fecal incontinence in primary care: prevalence, diagnosis, and health care utilization. Am J Obstet Gynecol.

[4] Tsuji I, Whalen S, Finucane TE (1995). Predictors of nursing home placement in community-based long-term care. J Am Geriatr Soc.

[5] Button D, Roe B, Webb C (1998). Consensus guidelines for the promotion and management of continence by primary health care teams: development, implementation and evaluation. NHS Executive Nursing Directorate. J Adv Nurs.

[6] Thekkinkattil DK, Lim M, Finan PJ (2008). Awareness of investigations and treatment of faecal incontinence among the general practitioners: a postal questionnaire survey. Colorectal Dis.

[7] Whitehead WE, Palsson OS, Simren M (2016). Treating fecal incontinence: an unmet need in primary care medicine. N C Med J.

[8] Ng K-S, Nassar N, Hamd K (2015). Prevalence of functional bowel disorders and faecal incontinence: an Australian primary care survey. Colorectal Dis.

[9] Johanson JF, Lafferty J (1996). Epidemiology of fecal incontinence: the silent affliction. Am J Gastroenterol.

[10] Kalantar JS, Howell S, Talley NJ (2002). Prevalence of faecal incontinence and associated risk factors; an underdiagnosed problem in the Australian community?. Med J Aust.

[11] Brown HW, Guan W, Schmuhl NB (2018). If we don't ask, they won't tell: Screening for urinary and fecal incontinence by primary care providers. J Am Board Fam Med.

[12] Delgado-Aros S, Solano Silveira R, Sala M (2011). Characteristics of primary care processes for the treatment of faecal incontinence in an urban area. Colorectal Dis.

[13] Helewa RM, Moloo H, Williams L (2017). Perspectives from patients and care providers on the management of fecal incontinence: a needs assessment. Dis Colon Rectum.

[14] Whitehead WE, Borrud L, Goode PS (2009). Fecal incontinence in US adults: epidemiology and risk factors. Gastroenterology.

[15] Quander CR, Morris MC, Melson J (2005). Prevalence of and factors associated with fecal incontinence in a large community study of older individuals. Am J Gastroenterol.

[16] Sultan AH, Kamm MA, Hudson CN (1993). Anal-sphincter disruption during vaginal delivery. N Engl J Med.

[17] National Institute for Health and Care Excellence (2014). Faecal incontinence in adults. Quality standard [QS54]. www.nice.org.uk/guidance/qs54.

[18] Rantell A, Dolan L, Bonner L (2016). Minimum standards for continence care in the UK. Neurourol Urodyn.

[19] The Royal Australian College of General Practitioners (2016). Curriculum for Australian general practice 2016 — CS16 core skills unit.

[20] The Royal Australian College of General Practitioners (2011). The RACGP curriculum for Australian general practice 2011.

[21] The Royal Australian College of General Practitioners (2016). Guidelines for preventive activities in general practice.

[22] Guinane J, Crone R (2018). Management of faecal incontinence. Aust J Gen Pract.

[23] The Royal Australian College of General Practitioners (2019). RACGP aged care clinical guide (Silver Book).

[24] Ribas Y, Coll M, Espina A (2017). Initiative to improve detection of faecal incontinence in primary care: the GIFT project. Fam Pract.

[25] Kunduru L, Kim SM, Heymen S, Whitehead WE (2015). Factors that affect consultation and screening for fecal incontinence. Clin Gastroenterol Hepatol.

